# Cardiovascular Health Changes in Young Adults and Risk of Later-Life Cardiovascular Disease

**DOI:** 10.1001/jamanetworkopen.2025.35573

**Published:** 2025-10-06

**Authors:** James W. Guo, Hongyan Ning, Norrina B. Allen, Amanda M. Perak, James M. Walker, Kelley Pettee Gabriel, Donald M. Lloyd-Jones

**Affiliations:** 1 Department of Medicine, Beth Israel Deaconess Medical Center, Harvard Medical School, Boston, Massachusetts; 2 Department of Preventive Medicine, Northwestern University Feinberg School of Medicine, Chicago, Illinois; 3 Department of Pediatrics, Lurie Children’s Hospital and Northwestern University Feinberg School of Medicine, Chicago, Illinois; 4 Department of Epidemiology, University of Alabama at Birmingham, Birmingham; 5 Framingham Center for Population and Prevention Science, Section of Preventive Medicine and Epidemiology, Department of Medicine, Boston University Chobanian & Avedisian School of Medicine, Boston, Massachusetts

## Abstract

**Importance:**

Associations of midlife cardiovascular health (CVH), measured once, with incident cardiovascular disease (CVD) are well described. Less is known about patterns of young adulthood CVH, including its changes and associations with later-life CVD outcomes.

**Objective:**

To model patterns of change in population-level and individual-level CVH through young adulthood and to assess whether they are associated with incident CVD in later life.

**Design, Setting, and Participants:**

The Coronary Artery Risk Development in Young Adults (CARDIA) study is a prospective longitudinal cohort study that enrolled Black and White participants at ages 18 to 30 years in 1985 and 1986 with subsequent follow-up examinations during the next 35 years at 4 urban US centers. Participants with at least 3 CVH measurements in young adulthood and subsequent follow-up with assessment of incident CVD events were included. Analyses were conducted from October 26, 2023, to May 15, 2024.

**Exposures:**

CVH was measured using the American Heart Association Life’s Essential 8 score. Patterns of CVH change in young adulthood (from examinations at years 0 to 20) were modeled with population-level trajectories and assessed by individual-level CVH status changes.

**Main Outcomes and Measures:**

Incident CVD (myocardial infarction, heart failure, stroke, coronary revascularization, and CVD death) after year 20.

**Results:**

There were 4241 participants in young adulthood (2354 [55.5%] female, 2042 [48.1%] self-identified as Black and 2199 [51.9%] self-identified as White) with a mean (SD) baseline age of 24.9 (3.6) years. In the trajectory analysis, 4 distinct CVH trajectory patterns were identified. Compared with the persistently high CVH trajectory, the moderate-to-low declining and moderate declining CVH trajectories had substantially higher risk for incident CVD. AHRs for incident CVD events ranged from 2.15 (95% CI, 1.04-4.47) in the persistently moderate pattern to 9.96 (95% CI, 4.75-20.86) in the moderate-to-low declining pattern. In the CVH status change analysis (n = 2857), compared with stable moderate CVH in young adulthood, stable high CVH had a lower risk (adjusted hazard ratio [AHR], 0.25 [95% CI, 0.09-0.69]), and stable low CVH had a higher risk (AHR, 5.91 [95% CI, 2.38-14.66]) for incident CVD. Each 10-point decrease in Life’s Essential 8 score between years 0 and 20 was associated with a 53% increase in CVD risk (AHR, 1.53 [95% CI, 1.31-1.78]).

**Conclusions and Relevance:**

In this prospective cohort study of young adults, unfavorable patterns of CVH change through young adulthood were associated with marked elevations in risk for incident CVD. These data suggest that achieving and maintaining high CVH throughout young adulthood through strategies of primordial prevention are important for prevention of later-life CVD.

## Key Points

**Question:**

Are longitudinal patterns of change in cardiovascular health (CVH) through young adulthood associated with risk for incident cardiovascular disease (CVD) in later life?

**Findings:**

In this cohort study of 4241 Black and White young adults, change in CVH was assessed at population and individual levels by trajectories and status change, respectively. Unfavorable longitudinal patterns were associated with an elevated risk for incident CVD as much as 9.96 times higher than favorable patterns.

**Meaning:**

These findings suggest that CVH in young adulthood is an important target for primordial prevention strategies to mitigate risk for future CVD.

## Teaser

This cohort study assesses the association between cardiovascular health trajectories and status changes in young adulthood and the risk for later-life clinical cardiovascular disease events among participants in the Coronary Artery Risk Development in Young Adults study.

## Introduction

Despite progress secured in past decades to reduce the burden of cardiovascular disease (CVD), it remains the leading cause of death in the US and worldwide.^1^ Data within the past 10 years indicate deceleration and reversal of declines in CVD morbidity and mortality.^2,3,4^ These trends may be further worsened in coming years by low prevalence of optimal cardiovascular health (CVH), particularly among young adults.^5,6,7,8^

Since the American Heart Association (AHA) introduced the construct of CVH defined by Life’s Simple 7 in 2010^9,10^ with a subsequent update in 2022 to Life’s Essential 8 (LE8),^11^ myriad studies have delineated the associations of CVH with cardiovascular outcomes.^1,10^ A growing body of research has begun to characterize CVH in young adulthood and to study its long-term associations with later-life subclinical^12,13^ and premature incident CVD.^6,7^ However, most prior studies assess CVH with a single, cross-sectional measure,^14,15,16,17^ and few have examined longitudinal patterns of CVH throughout young adulthood.^6,12,18^

There is increasing recognition of the importance of primordial prevention of CVD, particularly in children and young adults who may stand to benefit the most during the remaining life course from efforts to preserve, promote, and optimize CVH.^19,20,21^ Early intervention may serve to mitigate the cumulative deleterious impact of lower CVH during young adulthood.^18,19,22^ However, ideal CVH among young adults remains rare,^5,8^ and high CVH rarely persists throughout young adulthood, a period in the life course marked by changes in life circumstance and sharp declines in CVH.^12,23^

The association of patterns of change in CVH throughout young adulthood with later-life incident CVD remains unclear. We leveraged data from the Coronary Artery Risk Development in Young Adults (CARDIA) study to assess the association between CVH trajectories and CVH status changes in young adulthood on risk for later-life clinical CVD events, extending beyond prior work that largely examined subclinical end points. We used these 2 complementary approaches, as trajectory modeling enables study of CVH from a population perspective, whereas analysis of CVH status changes delineates CVH patterns at the individual level.

## Methods

### Study Sample

The CARDIA Study is a longitudinal cohort study that enrolled Black and White adults in 1985 and 1986. A total of 5115 healthy Black or White young adults aged 18 to 30 years were recruited in 1985 and 1986 from 4 urban centers: Birmingham, Alabama; Chicago, Illinois; Minneapolis, Minnesota; and Oakland, California. The cohort was approximately balanced within each center by race, sex, age, and educational level using community-based sampling. Periodic in-person examinations were completed for participants in 1987 to 1988 (year 2), 1990 to 1991 (year 5), 1992 to 1993 (year 7), 1995 to 1996 (year 10), 2000 to 2001 (year 15), 2005 to 2006 (year 20), 2010 to 2011 (year 25), 2015 to 2016 (year 30), and 2020 to 2022 (year 35). Participants were contacted every 6 months between examinations, with interval medical history ascertained annually by telephone interview. Detailed descriptions of the study design and conduct have been previously published.^24,25^ The study was approved by institutional review boards at all sites, and participants provided written informed consent at each examination. This study followed the Strengthening the Reporting of Observational Studies in Epidemiology (STROBE) reporting guideline.

### CVH Measurement and Quantification

We defined CVH according to the criteria established by the AHA LE8 score, including 4 health behaviors (diet, physical activity, smoking, and sleep) and 4 health factors (body mass index, non–high-density lipoprotein cholesterol level, blood glucose level, and blood pressure)^11^ described in eTable 1 in Supplement 1. Individual CVH metrics (each with a range of 0-100 points) were scored according to the AHA’s LE8 and the mean calculated to determine overall CVH (range, 0-100 points, with higher scores indicating better cardiovascular health) throughout young adulthood, which we defined using examination data from year 0 (mean age: 25 years) to year 20 (mean age: 45 years). Because sleep and hemoglobin A_1c_ data were not collected until the year 15 examination but are included in LE8, an imputation procedure that has been previously validated^26^ was used to impute values of these 2 variables at earlier examinations. Further details regarding measurement of individual LE8 metrics and the imputation procedure are provided in the eMethods in Supplement 1.

### CVD and Mortality Outcomes

The primary outcome was incident CVD, including myocardial infarction, heart failure, stroke, coronary revascularization, and CVD-related mortality occurring after the 20-year examination. Events were self-reported by participants during annual telephone interviews in which interviewers specifically inquired regarding hospitalizations and outpatient procedures. Deaths were identified via family contacts, internet searches, and regular queries of the National Death Index. The outcomes adjudication protocol is detailed in the eMethods in Supplement 1. Vital status follow-up is complete through 2020 (mean participant age, 60 years), marking 15 years after the 20-year examination.^27^

### Covariates

Self-reported demographic variables of age, sex, race, and maximal educational attainment were included as covariates for adjustment in all analyses. CARDIA enrolled participants who self-identified as Black or White, without separate identification of Hispanic ethnicity at baseline in 1985 and 1986. Subsequent examination data indicated that there was a small proportion who identified as Hispanic (n < 10). Race was self-reported and applied in this study as a social construct, without inherent biological meaning, to reflect an individual’s social determinants of health. Baseline LE8 score at year 0 was additionally included as an adjustment variable for the status change analysis and in a sensitivity analysis for trajectory.

### Statistical Analysis

Our study included 4241 participants for the trajectory analysis and a subset of 2857 participants for the status change analysis. Data were analyzed from October 26, 2023, to May 15, 2024. The characteristics of the excluded and included participants are detailed in the eMethods, eTable 2, and eFigure 1 in Supplement 1.

#### Trajectory Analysis

Trajectories of continuous LE8 scores (0-100 points) were modeled among CARDIA participants with complete LE8 data at 3 or more examinations between year 0 (mean age, 25 years) and year 20 (mean age, 45 years). We used the SAS procedure Proc Traj,^28,29^ which applied latent class modeling to identify subgroups of participants with similar long-term LE8 trajectories. Bayes information criteria and posterior probabilities were used to evaluate the fit of the most parsimonious trajectory model, choosing the best model as the one with the smallest negative Bayes information criteria. We assigned participants to the trajectory pattern for which they had the highest posterior predicted probability. Further details are provided in the eMethods and eTable 3 in Supplement 1.

#### Status Change Analysis

In a complementary analysis, we assessed change in CVH status (low, moderate, or high) by evaluating the direction of CVH status change (increasing, decreasing, or stable) using CVH data collected during the examinations at years 0 and 20. We followed the AHA scoring criteria to define low CVH status category as LE8 scores of 0 to 49, moderate as 50 to 79, and high as 80 to 100 points. Participants were classified as having increasing or decreasing CVH status change if their CVH status (defined by LE8 scores) crossed between at least 1 CVH category during young adulthood (between the examinations at years 0 and 20). Further details for these 2 subgroups are provided in eTable 4 in Supplement 1. Participants whose CVH did not cross categories and who maintained the same CVH category between years 0 and 20 were classified as having stable CVH. The stable moderate group was selected as the reference group as it represented the largest number of participants. Sensitivity analyses conducted using other reference groups yielded unstable estimates.

Furthermore, a comparison of continuous CVH scores was completed to examine the overall associated risk of incident CVD per 10-point decrease in LE8 scores between year 0 and year 20 examinations among participants included in the status change analysis. Additionally, hazard ratios (HRs) were estimated for each 10-point increase in LE8 score among participants in the increasing status change group and for each 10-point decrease in LE8 score among participants in the decreasing status change group.

We calculated descriptive statistics and crude incidence rates (per 1000 person-years) for the defined CVD outcomes across trajectory patterns and status change groups. Cox proportional hazards regression was used to estimate multivariable-adjusted HRs (AHRs) for associations of young adulthood CVH patterns with incident CVD outcomes after year 20. We performed additional subgroup analyses by race and sex. We furthermore assessed mean CVD-free years for each trajectory pattern and status change group by conducting an Irwin restricted mean survival time analysis^30^ truncated to the 15 years following the year 20 examination for which outcomes and vital status data have been collected. All analyses were performed using SAS, version 9.14 (SAS Institute Inc), with a 2-sided *P* < .05 considered statistically significant.

## Results

### Trajectory Analysis

#### Participant Characteristics

The trajectory analysis included 4241 CARDIA participants, with 2354 (55.5%) identifying as female and 1887 (44.5%) identifying as male, 2042 (48.1%) identifying as Black and 2199 (51.9%) identifying as White, and mean (SD) age of 24.9 (3.6) years at year 0 (Table 1). There were 903 participants (21.3%) in the persistently high CVH trajectory, 594 (65.8%) of whom were female and 309 (34.2%) of whom were male; 183 (20.3%) were Black and 720 (79.7%) were White. The mean (SD) baseline LE8 score was 87.4 (6.4) at year 0. Distributions of baseline LE8 scores for trajectory patterns are provided in eFigure 2 in Supplement 1.

**Table 1.** Baseline CARDIA Participant Characteristics by Trajectory Pattern

Characteristic	Mean (SD) CVH trajectory pattern				
	Overall (N = 4241)	Moderate-to-low declining (n = 382)	Moderate declining (n = 1357)	Persistently moderate (n = 1599)	Persistently high (n = 903)
Age, y	24.9 (3.6)	24.5 (3.7)	24.7 (3.7)	24.9 (3.6)	25.4 (3.4)
Sex, No. (%)					
Female	2354 (55.5)	225 (58.9)	689 (50.8)	846 (52.9)	594 (65.8)
Male	1887 (44.5)	157 (41.1)	668 (49.2)	753 (47.1)	309 (34.2)
Race, No. (%)					
Black	2042 (48.1)	262 (68.6)	848 (62.5)	749 (46.8)	183 (20.3)
White	2199 (51.9)	120 (31.4)	509 (37.5)	850 (53.2)	720 (79.7)
Total educational attainment, y	15.6 (2.6)	13.9 (2.3)	14.7 (2.4)	15.8 (2.5)	17.1 (2.3)
No. of CARDIA examinations attended	5.8 (1.4)	5.6 (1.4)	5.7 (1.4)	5.9 (1.3)	6.0 (1.3)
Baseline LE8 score^a^	75.2 (11.4)	57.0 (9.2)	68.7 (7.5)	77.7 (7.0)	87.4 (6.4)
HEI-2015 diet score^b^	61.9 (9.4)	56.4 (7.5)	58.4 (8.3)	62.1 (8.6)	69.3 (8.5)
Physical activity, min/wk	410.1 (362.1)	282.3 (327.1)	392.5 (367.5)	432.4 (370.1)	451.0 (339.4)
Current smoker, No. (%)	1188 (28.0)	252 (66.0)	601 (44.3)	312 (19.5)	23 (2.5)
BMI	24.4 (4.9)	29.6 (6.6)	25.8 (5.3)	23.4 (3.7)	21.9 (2.4)
Total cholesterol level, mg/dL	176.7 (32.9)	193.6 (36.6)	180.0 (34.2)	175.5 (31.6)	166.7 (27.3)
HDL cholesterol level, mg/dL	53.3 (13.1)	47.4 (12.7)	51.3 (12.9)	54.4 (13.0)	56.7 (12.1)
Fasting glucose level, mg/dL	82.0 (11.5)	85.0 (15.6)	82.8 (12.6)	81.3 (10.9)	81.0 (7.7)
Diabetes medication use, No. (%)	4 (0.1)	2 (0.5)	0	2 (0.1)	0
Systolic BP, mm Hg	110.2 (10.8)	115.6 (11.8)	111.9 (11.0)	109.2 (10.1)	107.0 (10.0)
Diastolic BP, mm Hg	68.4 (9.4)	71.5 (10.)	69.0 (10.1)	67.9 (8.9)	67.3 (8.3)
BP medication use, No. (%)	83 (2.0)	17 (4.5)	44 (3.2)	18 (1.1	4 (0.4)

Abbreviations: BMI, body mass index (calculated as weight in kilograms divided by height in square meters); BP, blood pressure; CARDIA, Coronary Artery Risk Development in Young Adults; CVH, cardiovascular health; HDL, high-density lipoprotein; HEI-2015, Healthy Eating Index 2015; LE8, Life’s Essential 8.

SI conversion factors: To convert glucose to mmol/L, multiply by 0.0555; HDL and total cholesterol to mmol/L, multiply by 0.0259.

^a^ Scores range from 0 to 100, with higher scores indicating better cardiovascular health.

^b^ Scores range from 0 to 100, with higher scores indicating better diet quality.

#### CVH Trajectory Patterns

Four distinct nonoverlapping CVH trajectory patterns were identified (Figure, A) from years 0 to 20 (mean age, 25-45 years). The 4 trajectories included a persistently high pattern that maintained high CVH throughout young adulthood (903 [21.3%]), a persistently moderate pattern (1599 [37.7%]), a moderate declining pattern (1357 [32.0%]), and a moderate-to-low declining pattern (382 [9.0%]). All 4 trajectory patterns observed declines in CVH during 20 years, with the moderate declining and moderate-to-low declining patterns showing more substantial declines.

**Figure.**
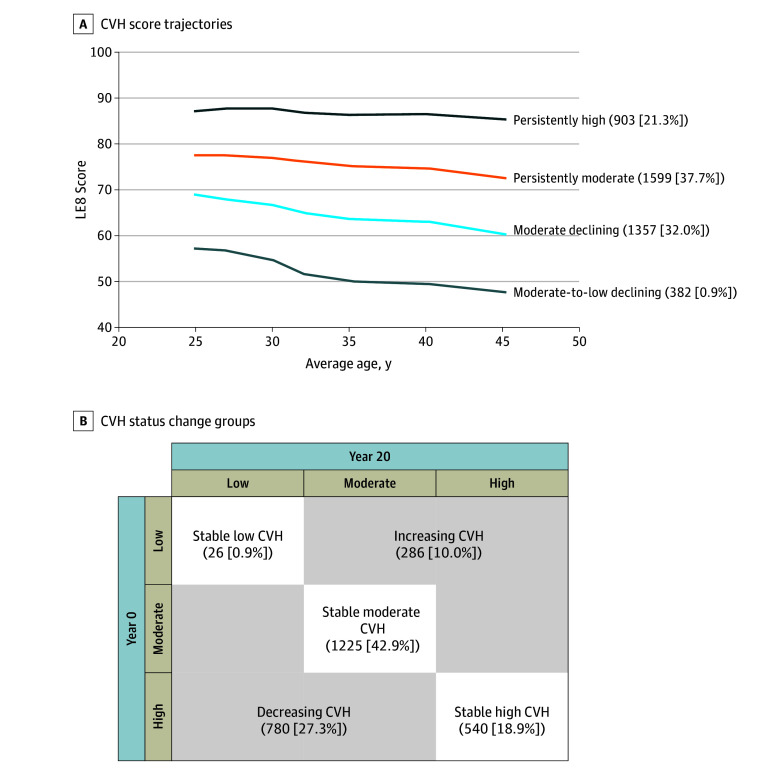
Young Adulthood Cardiovascular Health (CVH) Score Trajectories and CVH Status Change Groups LE8 indicates Life’s Essential 8 (range, 0-100, with higher scores indicating better CVH).

#### Rates of Incident CVD by CVH Trajectory Patterns

Table 2 and eFigure 3 in Supplement 1 present incidence rates by trajectory patterns. Among the participants in the trajectory analysis, there were 211 incident CVD events during a median follow-up time of 13.8 (range, 13.5-14.1) years after year 20 (median age, 58.8 [range, 58.5-59.1] years at first CVD event). The persistently high pattern had an incidence rate of 0.97 per 1000 person-years, while the moderate-to-low declining pattern had an incidence rate of 10.33 per 1000 person-years.

**Table 2.** CVD Incidence and Association of Cardiovascular Health Trajectory With CVD

Trajectory pattern	No. of participants (N = 4241)	CVD fatal and nonfatal events^a^		
		Crude incidence rate per 1000 person-years	AHR (95% CI)^b^	*P* value
Moderate-to-low declining	382	10.33	9.96 (4.75-20.86)	<.001
Moderate declining	1357	5.70	5.25 (2.69-10.26)	<.001
Persistently moderate	1599	2.30	2.15 (1.04-4.47)	.04
Persistently high	903	0.97	1 [Reference]	NA

Abbreviations: AHR, adjusted hazard ratio; CVD, cardiovascular disease; NA, not applicable.

^a^ Defined as myocardial infarction, heart failure, stroke, coronary revascularization, and cardiovascular death.

^b^ Adjusted for age, sex, race, and maximal educational attainment.

Incidence rates of CVD, including CVD event subtypes, are reported in eTable 5 and eFigure 4 in Supplement 1 for trajectory patterns. Incidence rates by sex and race subgroups that were calculated in sensitivity analyses for all trajectory patterns (eTables 6 and 7 in Supplement 1) demonstrated notable disparities in trajectory membership and event rates between subgroups.

#### Associations of Young Adult CVH Trajectories With Later-Life Incident CVD

In the trajectory analysis, with the persistently high trajectory set as the reference pattern, all other trajectory patterns of lower CVH through young adulthood demonstrated graded associations with significantly increased risk for incident CVD in later life (Table 2). AHRs for incident CVD events ranged from 2.15 (95% CI, 1.04-4.47) in the persistently moderate pattern to 9.96 (95% CI, 4.75-20.86) in the moderate-to-low declining pattern. In a sensitivity analysis, additional adjustment by baseline (year 0) LE8 score modestly attenuated the HRs, with the moderate declining and moderate-to-low declining patterns remaining associated with CVD outcomes (eTable 8 in Supplement 1). Furthermore, significant graded differences in mean (SD) CVD-free survival time during 15 years of follow-up were observed across trajectory patterns and ranged from 14.7 (0.1) years in the persistently high trajectory to 13.3 (0.2) years in the moderate-to-low declining trajectory (eFigure 5 in Supplement 1).

### Status Change Analysis

#### Participant Characteristics

The status change analysis was performed as a complement to the trajectory analysis, with the results presented separately. The status change analysis included 2857 CARDIA participants, with 1613 (56.5%) identifying as female and 1244 (43.5%) identifying as male, 1298 (45.4%) identifying as Black and 1559 (54.6%) identifying as White, and mean (SD) age of 25.0 (3.6) years at year 0 (Table 3). There were 540 participants (18.9%) in the stable high CVH status change group, with 356 (65.9%) female and 184 (34.1%) male, 106 (19.6%) Black and 434 (80.4%) White, and mean (SD) baseline LE8 score of 88.0 (5.1) at year 0.

**Table 3.** Baseline CARDIA Participant Characteristics by Status Change Group

Characteristic	CVH status change group					
	Overall (N = 2857)	Stable low CVH (n = 26)	Stable moderate CVH (n = 1225)	Stable high CVH (n = 540)	Decreasing CVH (n = 780)	Increasing CVH (n = 286)
Sex, No. (%)						
Female	1613 (56.5)	16 (61.5)	625 (51.0)	356 (65.9)	428 (54.9)	188 (65.7)
Male	1244 (43.5)	10 (38.5)	600 (49.0)	184 (34.1)	352 (45.1)	98 (34.3)
Race, No. (%)						
Black	1298 (45.4)	15 (57.7)	673 (54.9)	106 (19.6)	399 (51.2)	105 (36.7)
White	1559 (54.6)	11 (42.3)	552 (45.1)	434 (80.4)	381 (48.8)	181 (63.3)
Total educational attainment, mean (SD), y	15.8 (2.6)	13.2 (2.0)	15.3 (2.5)	17.2 (2.2)	15.6 (2.5)	16.3 (2.5)
No. of CARDIA examinations attended, mean (SD)	6.3 (1.1)	5.9 (1.4)	6.3 (1.1)	6.5 (1.0)	6.3 (1.1)	6.3 (1.1)
Age, mean (SD), y						
Year 0	25.0 (3.6)	26.0 (3.2)	25.1 (3.6)	25.4 (3.4)	24.8 (3.7)	24.8 (3.5)
Year 20	45.2 (3.6)	45.8 (3.2)	45.2 (3.7)	45.5 (3.4)	44.9 (3.7)	44.9 (3.5)
LE8 score, mean (SD)^a^						
Year 0	75.7 (11.3)	43.1 (5.7)	69.4 (7.0)	88.0 (5.1)	79.9 (10.5)	70.4 (9.5)
Year 20	69.9 (14.6)	38.0 (8.4)	65.1 (8.1)	87.9 (5.6)	61.2 (13.6)	83.3 (9.4)
HEI-2015 diet score, mean (SD)^b^						
Year 0	62.1 (9.4)	56.6 (6.8)	58.6 (8.2)	68.6 (8.4)	63.7 (9.5)	60.5 (8.1)
Year 20	69.8 (10.5)	61.2 (9.1)	67.2 (10.0)	77.5 (8.2)	67.1 (9.7)	74.5 (9.4)
Physical activity, mean (SD), min/wk						
Year 0	405.5 (360.8)	98.3 (196.7)	390.3 (380.8)	463.8 (334.5)	430.4 (343.6)	321.2 (342.8)
Year 20	324.5 (339.0)	125.3 (212.0)	323.2 (345.2)	421.6 (325.6)	237.3 (323.9)	403.4 (314.3)
Current smoker, No. (%)						
Year 0	723 (25.3)	20 (76.9)	457 (37.3)	16 (3.0)	150 (19.2)	80 (28.0)
Year 20	537 (18.8)	19 (73.1)	318 (26.0)	7 (1.3)	175 (22.4)	18 (6.3)
BMI, mean (SD)						
Year 0	24.2 (4.7)	32.1 (6.6)	25.1 (4.9)	21.8 (2.3)	24.4 (4.4)	23.6 (5.3)
Year 20	29.2 (6.8)	34.8 (8.1)	30.6 (6.7)	24.4 (3.3)	31.2 (6.8)	26.1 (5.8)
Total cholesterol level, mean (SD), mg/dL						
Year 0	177.1 (32.5)	217.5 (29.6)	181.5 (33.3)	166.9 (27.4)	173.0 (29.9)	185.0 (35.9)
Year 20	185.7 (34.5)	209.1 (35.0)	188.1 (35.3)	175.3 (28.2)	190.8 (35.7)	178.7 (32.8)
HDL cholesterol level, mean (SD), mg/dL						
Year 0	53.5 (12.9)	42.4 (13.3)	52.4 (12.7)	56.5 (12.3)	52.9 (12.8)	55.6 (13.0)
Year 20	54.3 (16.7)	42.9 (11.6)	52.2 (15.5)	61.7 (17.4)	50.8 (15.7)	60.5 (17.2)
Fasting glucose level, mean (SD), mg/dL						
Year 0	81.9 (11.4)	96.1 (33.5)	82.5 (12.2)	80.6 (7.2)	81.7 (11.3)	81.2 (8.8)
Year 20	97.2 (25.1)	145.7 (80.2)	98.0 (23.2)	90.4 (8.1)	101.4 (31.4)	90.9 (15.1)
Diabetes medication use, No. (%)						
Year 0	5 (0.2)	2 (7.7)	1 (0.1)	0	2 (0.3)	0
Year 20	102 (3.6)	8 (30.8)	45 (3.7)	1 (0.2)	44 (5.6)	4 (1.4)
Systolic BP, mean (SD), mm Hg						
Year 0	109.8 (10.7)	117.5 (11.8)	111.3 (10.9)	106.3 (9.6)	109.9 (10.5)	109.0 (10.6)
Year 20	115.3 (14.3)	127.9 (22.1)	117.3 (13.7)	108.2 (9.9)	118.7 (15.9)	109.8 (11.2)
Diastolic BP, mean (SD), mm Hg						
Year 0	68.4 (9.3)	75.5 (11.4)	68.8 (9.8)	66.9 (8.3)	68.4 (8.9)	68.8 (9.6)
Year 20	71.9 (11.0)	80.4 (13.3)	73.6 (10.3)	65.1 (7.9)	75.3 (11.9)	67.6 (8.8)
BP medication use, No. (%)						
Year 0	53 (1.9)	2 (7.7)	36 (2.9)	2 (0.4)	9 (1.2)	4 (1.4)
Year 20	460 (16.1)	13 (50.0)	243 (19.8)	15 (2.8)	170 (21.8)	19 (6.6)

Abbreviations: BMI, body mass index (calculated as weight in kilograms divided by height in square meters); BP, blood pressure; CARDIA, Coronary Artery Risk Development in Young Adults; CVH, cardiovascular health; HDL, high-density lipoprotein; HEI-2015, Healthy Eating Index 2015; LE8, Life’s Essential 8.

SI conversion factors: To convert glucose to mmol/L, multiply by 0.0555; HDL and total cholesterol to mmol/L, multiply by 0.0259.

^a^ Scores range from 0 to 100, with higher scores indicating better cardiovascular health.

^b^ Scores range from 0 to 100, with higher scores indicating better diet quality.

Distributions of baseline LE8 scores for status change groups are provided in eFigure 6 in Supplement 1. Comparison of individual LE8 metric scores at years 0 and 20 are provided in eTable 9 in Supplement 1.

#### Status Change Groups

Five status change groups were assigned (Figure, B), including a stable high CVH group that had high CVH at both years 0 and 20 (540 [18.9%]), a stable moderate CVH group (1225 [42.9%]), a stable low CVH group (26 [0.9%]), an increasing CVH group (286 [10.0%]), and a decreasing CVH group (780 [27.3%]). Participants were included in the increasing or decreasing CVH groups if their CVH status crossed at least 1 category between years 0 and 20. For example, the increasing group predominantly consisted of participants who had moderate CVH at year 0 and subsequently had high CVH at year 20 (eTable 4 in Supplement 1).

#### Rates of Incident CVD by Status Change Groups

Table 4 and eFigure 3 in Supplement 1 present incidence rates by status change groups. Among the participants in the status change analysis, there were 142 incident CVD events during a median follow-up time of 13.8 (range, 13.5-14.1) years after year 20 (median age, 58.8 [range, 58.5-59.1] years at first CVD event). The stable high CVH group had an incidence rate of 0.77 per 1000 person-years while the stable low CVH group had an incidence rate of 26.79 per 1000 person-years, though the latter had a small number of participants.

**Table 4.** CVD Incidence and Association Between Status Change With CVH

CVH status change group	No. of participants (n = 2857)	CVD fatal and nonfatal events^a^		
		Crude incidence rate per 1000 person-years	AHR (95% CI)^b^	*P* value
Stable low CVH	26	26.79	5.91 (2.38-14.66)	<.001
Stable high CVH	540	0.77	0.25 (0.09-0.69)	.007
Increasing CVH^c^	286	3.43	1.04 (0.53-2.04)	.90
Decreasing CVH^c^	780	5.03	1.36 (0.90-2.06)	.14
Stable moderate CVH	1225	3.97	1 [Reference]	NA
Continuous CVH score (per 10-point lower LE8 between years 0 and 20)^d^	NA	NA	1.53 (1.31-1.78)	<.001

Abbreviations: AHR, adjusted hazard ratio; CVD, cardiovascular disease; CVH, cardiovascular health; LE8, Life’s Essential 8; NA, not applicable.

^a^ Defined as myocardial infarction, heart failure, stroke, coronary revascularization, and cardiovascular death.

^b^ Adjusted for age, sex, race, and maximal educational attainment.

^c^ Increasing and decreasing CVH groups defined as participants whose CVH crossed over 1 or more categories, defined as high CVH (80-100), moderate CVH (50-79), and low CVH (0-49).

^d^ Adjusted for age, sex, race, maximal educational attainment, and baseline (year 0) Life’s Essential 8 score.

Incidence rates of CVD, including by CVD event subtypes, are reported in eTable 10 and eFigure 7 in Supplement 1 for status change groups. Incidence rates of CVD events by sex and race were calculated in sensitivity analyses for all status change groups in eTables 6 and 7 in Supplement 1, demonstrating notable disparities in status change group membership (prominently in the stable high CVH group) and event rates between subgroups.

#### Associations of Young Adult CVH Status Change With Later-Life Incident CVD

In the CVH status change analysis, the stable moderate group was set as the reference group as it included the largest number of participants. The stable high group had an AHR of 0.25 (95% CI, 0.09-0.69), while the stable low group had an AHR of 5.91 (95% CI, 2.38-14.66) for incident CVD. Notably, the increasing and decreasing groups did not demonstrate significantly different AHRs compared with the stable moderate group (Table 4). Furthermore, in comparing mean (SD) CVD-free survival time, the stable high group experienced 14.8 (0.1) years and the stable low group experienced 11.6 (1.0) years, which were significantly different than the 14.1 (0.1) by the stable moderate reference group (eFigure 5 in Supplement 1).

In assessment of change in continuous CVH score between years 0 and 20, each 10-point decrease in LE8 score was overall associated with a 53% increased risk (HR, 1.53 [95% CI, 1.31-1.78]) for incident CVD (Table 4). Changes in continuous CVH score were also assessed for participants in the increasing and decreasing groups (per 10-point increase or decrease in LE8 score, respectively). While no significant difference in hazards was observed for each 10-point increase in LE8 score in the increasing group, each 10-point decrease in LE8 score between years 0 and 20 in the decreasing group had an HR of 1.95 (95% CI, 1.38-2.75; *P* < .001).

## Discussion

In this cohort study of longitudinal CVH changes through young adulthood, we demonstrated several significant findings using 2 complementary approaches. First, our trajectory analysis enabled us to study CVH patterns at the population level. We observed 4 distinct CVH trajectories during 20 years of follow-up through young adulthood. Compared with the persistently high pattern, each less-favorable CVH trajectory demonstrated both a higher incidence of CVD events and a stepwise increase in later-life CVD risk. Notably, all 4 trajectory patterns started and remained separated without convergence, suggesting that CVH trajectories may already be established by 25 years of age, aligning with prior studies that show significant declines in CVH may begin as early as childhood or adolescence.^23,31^

Second, in the CVH status change analysis, we assigned 5 status change groups to delineate CVH patterns at the individual level. Compared with the stable moderate group, the stable low group had significantly elevated incidence of and risk for later-life CVD; the stable high group had significantly lower incidence and risk for CVD; and neither the increasing nor decreasing group differed significantly, perhaps indicating deleterious residual cumulative or legacy effect of lower CVH status experienced at any point in young adulthood.^18^ Assessment of continuous CVH scores quantified the significantly higher risk for incident CVD associated with each 10-point decrease in LE8 from years 0 to 20.

Overall, these data imply that improvement and/or maintenance of high CVH through the critical period of young adulthood, achieved through strategies of primordial prevention, may substantially reduce later-life CVD. Our findings strongly reinforce and extend prior evidence showing that high CVH in young adulthood, conceptualized both as a single measurement^7^ and as a cumulative exposure,^18,32^ is associated with exceedingly low rates of later-life premature CVD and mortality. We found notable disparities in young adulthood CVH trends and CVD outcomes by sex and race, underscoring the urgent need for interventions to mitigate such inequities.^1,33,34,35^ In short, the current observations indicate that change matters: improvements in CVH can decrease future risk, and the earlier high CVH is attained and maintained, the better.

There is growing recognition that patterns of CVH change in young adulthood may influence cardiovascular outcomes. Improvement in CVH through young adulthood has been associated with lower risk for CVD events.^6^ Furthermore, trajectories of CVH modeled from childhood through adulthood delineate substantial CVH declines through young adulthood and elevated risk for subclinical atherosclerosis associated with less favorable CVH trajectories.^12^ CVH does not remain static; rather, it fluctuates throughout the life course, as 80% of young adults with ideal CVH lose that ideal CVH profile by middle age.^36^ Such CVH decline is particularly prominent during the young adulthood period for reasons that may be shaped by the young adulthood milieu, including social and developmental transitions (eg, psychosocial stressors, aging out of parental insurance leading to interrupted care continuity).^23^ Notably, adolescents with normal body mass index are twice as likely to have optimal CVH later in young adulthood compared with those who have obesity, underscoring the longitudinal importance of sustaining optimal health and lifestyle starting from early life.^37^ Although previous studies have begun to quantify the associations of CVH in young adulthood with various clinical or subclinical outcomes,^14,15,31,38,39,40,41^ none have assessed longitudinal patterns of CVH change and their relationships with clinical CVD events. This study is the first, to our knowledge, to implement complementary analyses to assess CVH patterns of change through young adulthood at both a population and an individual level and their associations with clinical CVD events.

Numerous studies have described the benefits of achieving or preserving high CVH into midlife, including greater longevity and health span,^42^ compression of morbidity,^43^ lower rates of CVD^44^ and other chronic diseases of aging,^39,45,46^ and higher quality of life with lower health care expenditure,^40,47,48^ among others.^1,5,11,19^ Low CVH has been associated with poor outcomes,^49,50,51,52^ with 70% of CVD events attributable to low and moderate CVH levels.^53^ Despite the recognized importance of CVH, high CVH is rare^54^ (1 in 4 US young adults), representing a significant public health concern.^8,53^ Unfortunately, this issue has not improved during the past decade^8,55^ and may be further exacerbated by an overall low awareness of prevalent, notable cardiovascular risk factors (eg, obesity, hypertension, hyperlipidemia, diabetes) among young adults.^56^

However, favorable changes in CVH behaviors and factors can reduce the likelihood of clinical or subclinical CVD development.^57,58^ Targeted interventions in young adulthood and earlier in the life course to promote CVH may yield substantial gains in long-term health.^36,53^ For example, early-life lifestyle intervention trials, including the SI! Program for Secondary Schools^59^ and the Special Turku Coronary Risk Factor Intervention Project (STRIP),^60^ have actualized primordial prevention strategies, underscoring implementation challenges and the need for age-specific interventions.^61,62^ In the STRIP trial, participants randomized during infancy to receive biannual, heart-healthy dietary and lifestyle counseling through 20 years of age experienced significantly higher CVH and lower subclinical CVD burden by adolescence than participants in the control arm,^16^ a finding that persisted on reassessment of CVH years later in young adulthood.^22^ Ultimately, promoting high CVH in all adults, with even partial improvement in CVH scores, may yield substantial reductions in CVD event rates,^53^ especially among young adults who stand to benefit the most with respect to cumulative exposures to CVH levels during the remainder of their life course.^63,64^

### Limitations

This study has some potential limitations to note. Given the young baseline age of CARDIA participants, only a limited number of premature events have been captured thus far through follow-up until the mean age of 60 years. This may have contributed to the null findings in the increasing and decreasing status change groups. Additionally, the longitudinal nature of the CARDIA cohort predisposes to participant attrition bias, most often with participants most at risk for incident CVD having inconsistent follow-up examinations. Despite the relatively small number of observed events and possible attrition bias, we nevertheless detected significant differences in risk among multiple other patterns and groups, underscoring the durability of our findings, which reinforces the importance of CVH in young adulthood. With longer follow-up and more outcome events, we may observe additional statistical significance not evident in the present study. There was a small number of participants in the stable low CVH group, potentially leading to imprecise results. Because participants who developed CVD prior to year 20 were excluded, the magnitude of the reported HRs may actually be underestimated. Despite judicious selection of covariates, residual confounding may persist. Though included participants had a mean age of 24.9 years at baseline, CVH trajectories were already established and distinct, without overlap during 20 years of follow-up. Future studies may investigate further upstream in the life course to assess convergences of CVH trajectories and study their associations with later-life CVD events.

## Conclusions

In this prospective, longitudinal cohort study, unfavorable patterns of CVH change through 20 years of young adulthood were associated with marked elevations in risk for later-life incident CVD. Our findings emphasize the importance of CVH in the young adulthood period for future cardiovascular outcomes and may support clinical guideline and policy initiatives to promote, maintain, or restore optimal CVH at both an individual and a population level through strategies of primordial prevention to mitigate risk for future CVD.

## Notes

Supplement 1. **eMethods.** Participant Inclusion, LE8 Imputation, and Trajectory Analysis **eFigure 1.** Flow Diagram of Included Participants From the CARDIA Study Sample **eFigure 2.** Distribution of Baseline LE8 Scores by CVH Trajectory Patterns **eFigure 3.** Incidence Rates of Cardiovascular Disease **eFigure 4.** Event Incidence Rates by Trajectory Patterns **eFigure 5.** Restricted Mean Survival Time Analysis: Mean CVD-Free Survival and Survival After CVD Event by Trajectory Patterns and Status Change Groups **eFigure 6.** Distribution of Baseline LE8 Scores by CVH Status Change Groups **eFigure 7.** Event Incidence Rates by Status Change Groups **eTable 1.** Life’s Essential 8 Score Components **eTable 2.** Included and Excluded CARDIA Participant Characteristics at Baseline **eTable 3.** Adjusted Hazard Ratios of Incident CVD Events by CVH Trajectory Patterns Excluding Individuals With Mean PPP<0.7 **eTable 4.** Distribution of Status Change Groups (Increasing vs Decreasing) **eTable 5.** Incidence Rates of CVD Events Occurring During Follow-Up, by Trajectory Pattern **eTable 6.** Sensitivity Analysis: CVD Incidence by CVH Trajectories and CVH Status Change in Young Adulthood, by Sex **eTable 7.** Sensitivity Analysis: CVD Incidence by CVH Trajectories and CVH Status Change in Young Adulthood, by Race **eTable 8.** CVD Incidence and Hazard Ratios of Incident CVD by CVH Trajectories with Adjustment by Baseline LE8 Score **eTable 9.** Individual LE8 Metric Scores at Years 0 and 20, by Status Change Group **eTable 10.** Incidence Rates of CVD Events Occurring During Follow-Up, by Status Change Group
eReferences

Supplement 2.
Data Sharing Statement
