# Supplementary material for: Cardiovascular Health Changes in Young Adults and Risk of Later-Life Cardiovascular Disease
Source: JAMA Netw Open. 2025 Oct 6;8(10):e2535573. doi: 10.1001/jamanetworkopen.2025.35573 (PMC12501802; doi:10.1001/jamanetworkopen.2025.35573)
Supplement: Supplement 1. — eMethods. Participant Inclusion, LE8 Imputation, and Trajectory Analysis eFigure 1. Flow Diagram of Included Participants From the CARDIA Study Sample eFigure 2. Distribution of Baseline LE8 Scores by CVH Trajectory Patterns eFigure 3. Incidence Rates of Cardiovascular Disease eFigure 4. Event Incidence Rates by Trajectory Patterns eFigure 5. Restricted Mean Survival Time Analysis: Mean CVD-Free Survival and Survival After CVD Event by Trajectory Patterns and Status Change Groups eFigure 6. Distribution of Baseline LE8 Scores by CVH Status Change Groups eFigure 7. Event Incidence Rates by Status Change Groups eTable 1. Life’s Essential 8 Score Components eTable 2. Included and Excluded CARDIA Participant Characteristics at Baseline eTable 3. Adjusted Hazard Ratios of Incident CVD Events by CVH Trajectory Patterns Excluding Individuals With Mean PPP<0.7 eTable 4. Distribution of Status Change Groups (Increasing vs Decreasing) eTable 5. Incidence Rates of CVD Events Occurring During Follow-Up, by Trajectory Pattern eTable 6. Sensitivity Analysis: CVD Incidence by CVH Trajectories and CVH Status Change in Young Adulthood, by Sex eTable 7. Sensitivity Analysis: CVD Incidence by CVH Trajectories and CVH Status Change in Young Adulthood, by Race eTable 8. CVD Incidence and Hazard Ratios of Incident CVD by CVH Trajectories with Adjustment by Baseline LE8 Score eTable 9. Individual LE8 Metric Scores at Years 0 and 20, by Status Change Group eTable 10. Incidence Rates of CVD Events Occurring During Follow-Up, by Status Change Group eReferences [file jamanetwopen-e2535573-s001.pdf]

Supplementary Online Content

Guo JW, Ning H, Allen NB, et al. Cardiovascular health changes in young adults and risk of later-life cardiovascular disease. *JAMA Netw Open.* 2025;8(10):e2535573. doi:10.1001/jamanetworkopen.2025.35573

eMethods. Participant Inclusion, LE8 Imputation, and Trajectory Analysis
eFigure 1. Flow Diagram of Included Participants From the CARDIA Study Sample
eFigure 2. Distribution of Baseline LE8 Scores by CVH Trajectory Patterns
eFigure 3. Incidence Rates of Cardiovascular Disease
eFigure 4. Event Incidence Rates by Trajectory Patterns
eFigure 5. Restricted Mean Survival Time Analysis: Mean CVD-Free Survival and Survival After CVD Event by Trajectory Patterns and Status Change Groups
eFigure 6. Distribution of Baseline LE8 Scores by CVH Status Change Groups
eFigure 7. Event Incidence Rates by Status Change Groups
eTable 1. Life's Essential 8 Score Components
eTable 2. Included and Excluded CARDIA Participant Characteristics at Baseline
eTable 3. Adjusted Hazard Ratios of Incident CVD Events by CVH Trajectory Patterns Excluding Individuals With Mean PPP <0.7
eTable 4. Distribution of Status Change Groups (Increasing vs Decreasing)
eTable 5. Incidence Rates of CVD Events Occurring During Follow-Up, by Trajectory Pattern
eTable 6. Sensitivity Analysis: CVD Incidence by CVH Trajectories and CVH Status Change in Young Adulthood, by Sex
eTable 7. Sensitivity Analysis: CVD Incidence by CVH Trajectories and CVH Status Change in Young Adulthood, by Race
eTable 8. CVD Incidence and Hazard Ratios of Incident CVD by CVH Trajectories with Adjustment by Baseline LE8 Score
eTable 9. Individual LE8 Metric Scores at Years 0 and 20, by Status Change Group
eTable 10. Incidence Rates of CVD Events Occurring During Follow-Up, by Status Change Group
eReferences

This supplementary material has been provided by the authors to give readers additional information about their work.

© 2025 Guo JW et al. *JAMA Network Open*.

eMethods. Participant Inclusion, LE8 Imputation, and Trajectory Analysis

CVH Definition and Assessment

Dietary habits were assessed using the CARDIA Diet History questionnaire[1,2], which gathered data on general eating patterns, and were assessed according to the Healthy Eating Index 2015[3] to create a diet score that evaluates adherence to the dietary guidelines recommended for Americans. Physical activity was assessed using the validated CARDIA Physical Activity History questionnaire, which recorded the time spent weekly in 13 different physical activities over a year.[4] Smoking habits were self-reported through an interviewer-led questionnaire. Sleep was assessed by self-reported average hours of sleep per night. Because sleep data was only collected after the Y15 exam, the sleep score was imputed according to the procedure detailed in the "LE8 Imputation Procedure" section below and which has been previously used and validated in other studies.[5]

Body mass index (BMI) was calculated by dividing a person's weight (kg) by their height squared (m^2) using standard measurements from trained technicians. Study participants were instructed to fast for 12 hours and to avoid tobacco use or physical exercise for two hours prior to each medical examination. Fasting venous blood samples were then collected in the morning. Total and HDL cholesterol levels were measured using standard enzymatic methods with the ABA Biochromatic instrument. Glucose levels were determined through a standard hexokinase method. Blood pressure was measured after 5 minutes seated at rest by trained technicians using a random zero sphygmomanometer until Year 20, and using oscillometric machines (Omron HEM-970XL) thereafter, with the average of the second and third readings reported.[6]

LE8 Imputation Procedure

To calculate the LE8 score, we included the following factors per AHA criteria: diet quality, physical activity, nicotine exposure, sleep duration, blood pressure, BMI, non-HDL cholesterol levels, fasting glucose, glycated hemoglobin (HbA1c), and medication treatment. Sleep and HbA1c data were not collected till exam Year 15 and Year 20, respectively, and were thus considered "missing" in prior exams. Excluding participants with missing data from analyses results in loss of statistical power and may give rise to selection bias.

To overcome this, we multiply imputed missing sleep and HbA1c in R software using multivariate imputation by chained equations (MICE) with a method adapted to the multilevel structure of the data and continuous nature of the variable ("2l.pan"). The participants identification number was used as the class variable to specify that the repeated measures belong to the same participant. Demographics, social economic status, lifestyle, and clinical factors were included in the imputation model. Convergence of the chained equation procedure was visually evaluated from trace plots of the mean and standard deviation of the imputed data against the iteration numbers and no visible strong trends for any imputed variables were observed. Each regression analysis was performed separately on each of the 50 imputed datasets, and the results were combined using Rubin's rules.[7] This imputation method was previously validated, reviewed, and approved by the CARDIA Data and Analysis Committee.

© 2025 Guo JW et al. *JAMA Network Open*.

CVD and Mortality Outcomes Adjudication Protocol

Reported events were verified and adjudicated by 2 clinician members of the CARDIA Endpoints Surveillance and Adjudication Subcommittee through medical record review using standard clinical definitions, with disagreements adjudicated by the Subcommittee.

Description of Included and Excluded Participants

We excluded 1 participant who withdrew consent, 2 participants who had undergone gender transition, 270 participants who developed any CVD endpoints prior to Y20, and 601 participants with fewer than 3 LE8 scores between Y0 and Y20 (requisite to model trajectory patterns).

Thus, for the trajectory pattern, we included 4,241 participants who attended ≥3 examinations between Y0 and Y20. For the status change analysis sample, we included 2,857 participants after excluding an additional 1,384 participants who were missing an LE8 score at Y0 and/or Y20 (requisite to determine status change groups).

The two analyses have different sample sizes; we required participants to attend 3 exams between Y0 and Y20 to be included in our trajectory analysis (trajectories can be modeled with a minimum of 3 data points). However, for the status change analysis, by definition, only participants who were examined at *both* Y0 and Y20 were included. As such, a number of participants who met criteria for inclusion in the trajectory analysis were not included in the status change analysis because they did not present for follow-up examination at Y20.

To note, in this study we do not draw direct comparison between the trajectory and status change analyses. Rather, they complement one another.

Trajectory Analysis: Posterior Predicted Probability (PPP)

Our trajectory model had PPPs ranging from 0.89-0.93. We conducted an additional sensitivity analysis by excluding individuals (N=385) with mean PPP<0.7, and the associations remained unchanged (**eTable 3**). We therefore present the full results from the trajectory analyses.

© 2025 Guo JW et al. *JAMA Network Open*.

eFigure 1. Flow Diagram of Included Participants From the CARDIA Study Sample

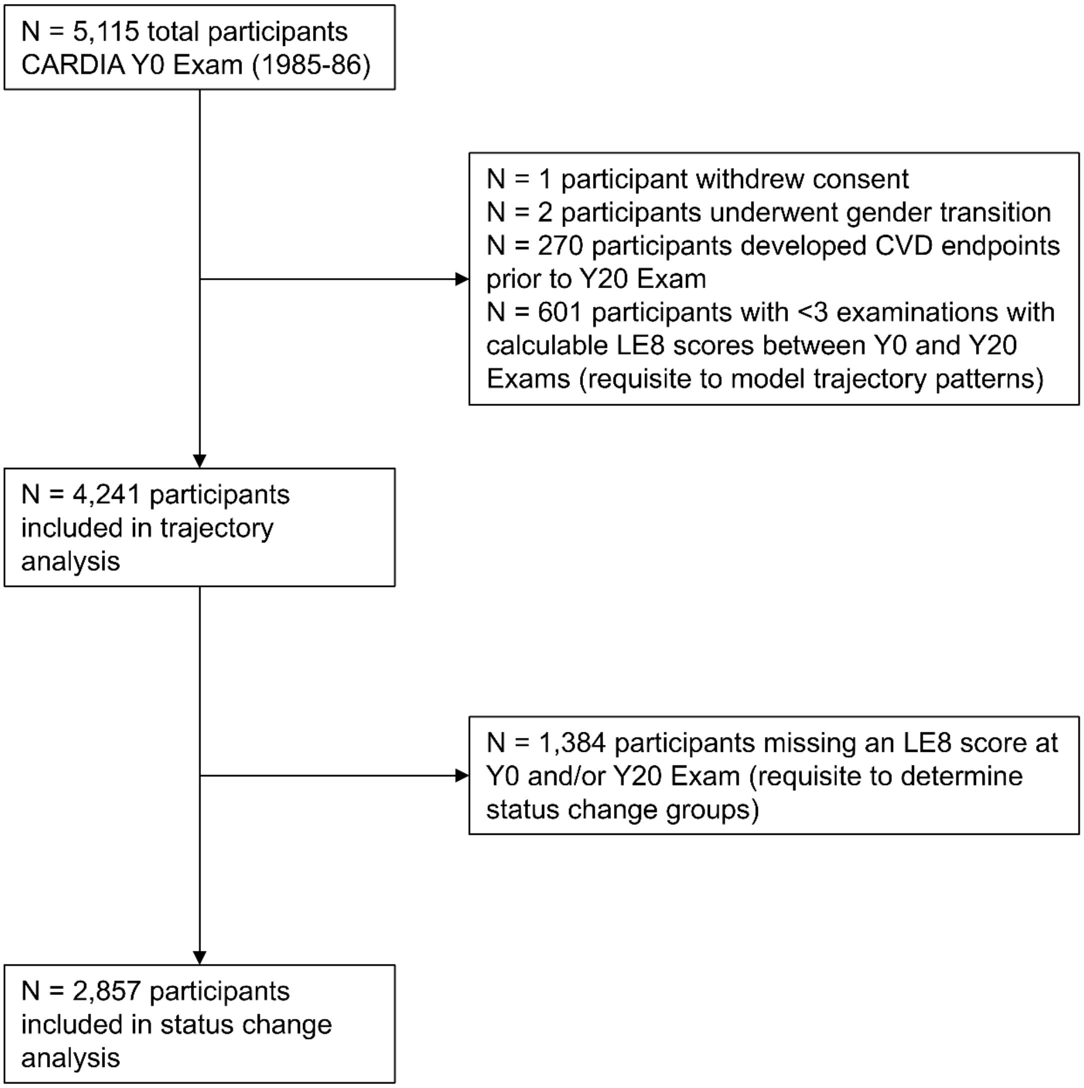

© 2025 Guo JW et al. *JAMA Network Open*.

eFigure 2. Distribution of Baseline LE8 Scores by CVH Trajectory Patterns

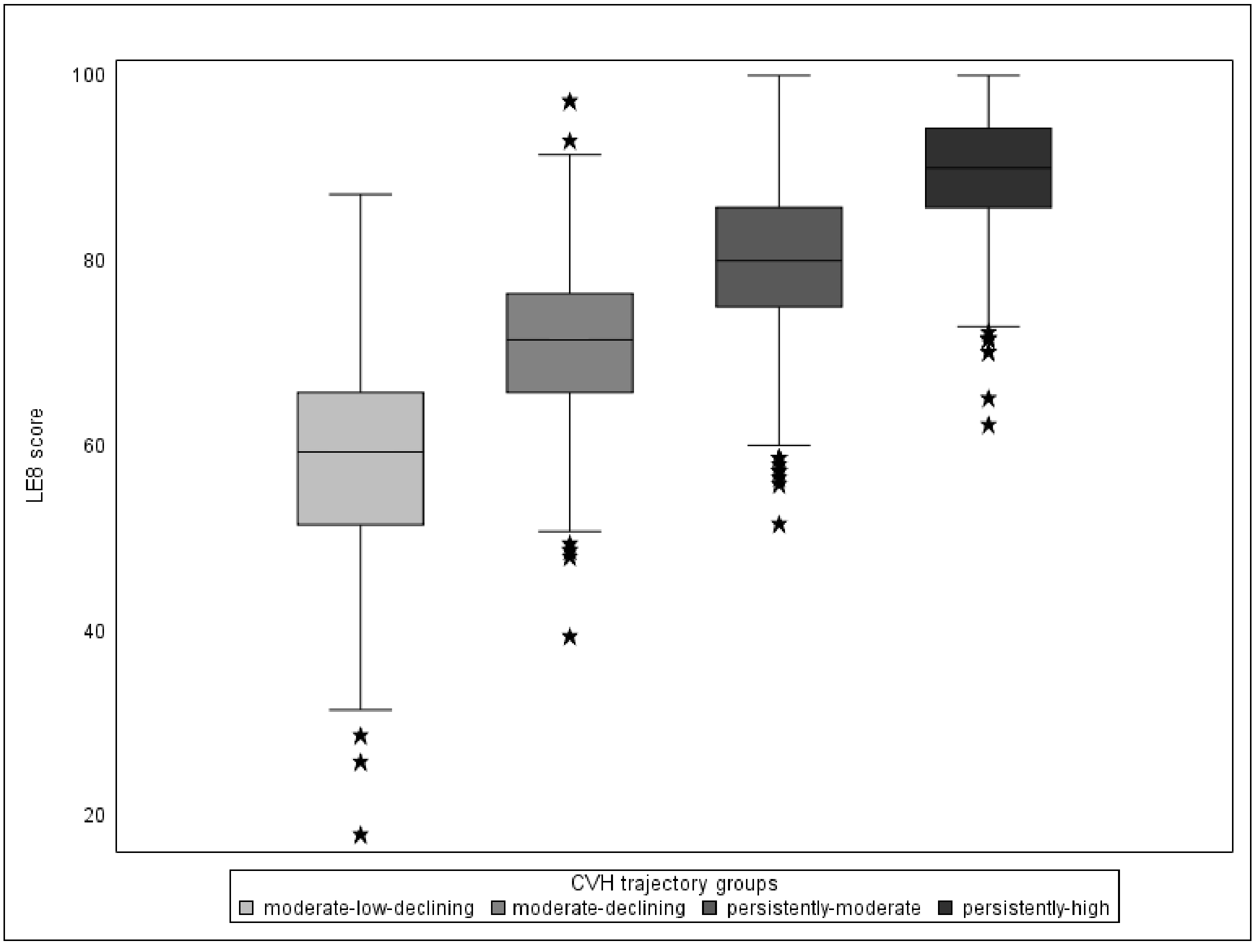

© 2025 Guo JW et al. *JAMA Network Open*.

eFigure 3. Incidence Rates of Cardiovascular Disease

(A) Trajectory Patterns and **(B)** Status Change Groups

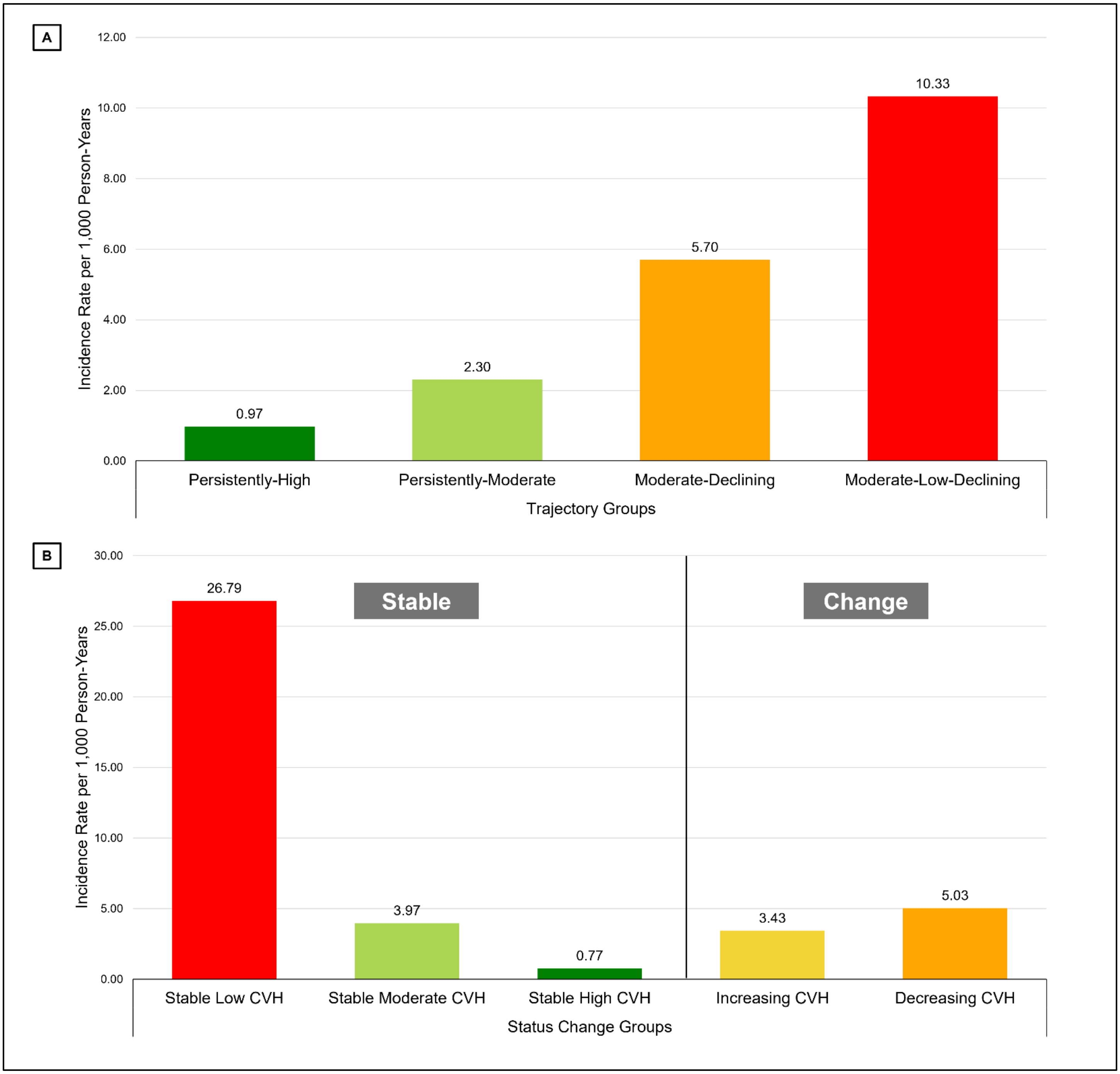

© 2025 Guo JW et al. *JAMA Network Open*.

eFigure 4. Event Incidence Rates by Trajectory Patterns

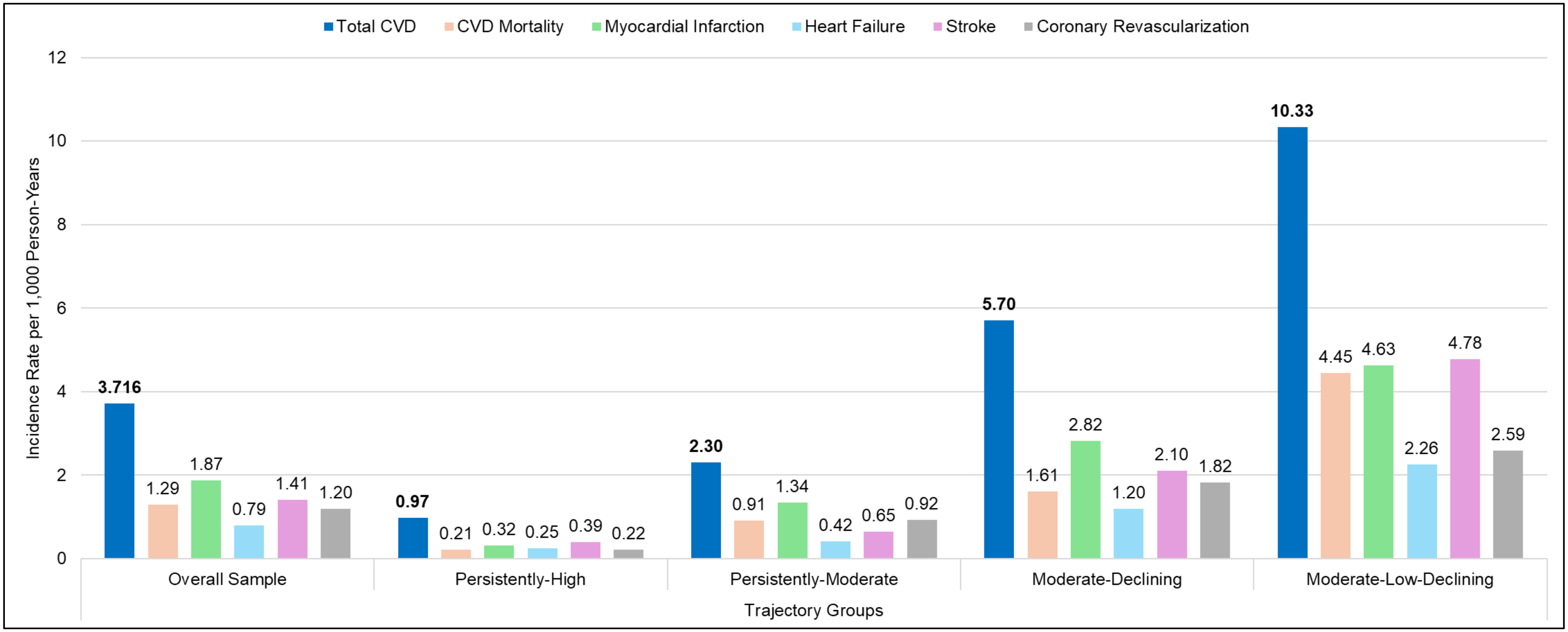

© 2025 Guo JW et al. *JAMA Network Open*.

eFigure 5. Restricted Mean Survival Time Analysis: Mean CVD-Free Survival and Survival After CVD Event by Trajectory Patterns and Status Change Groups

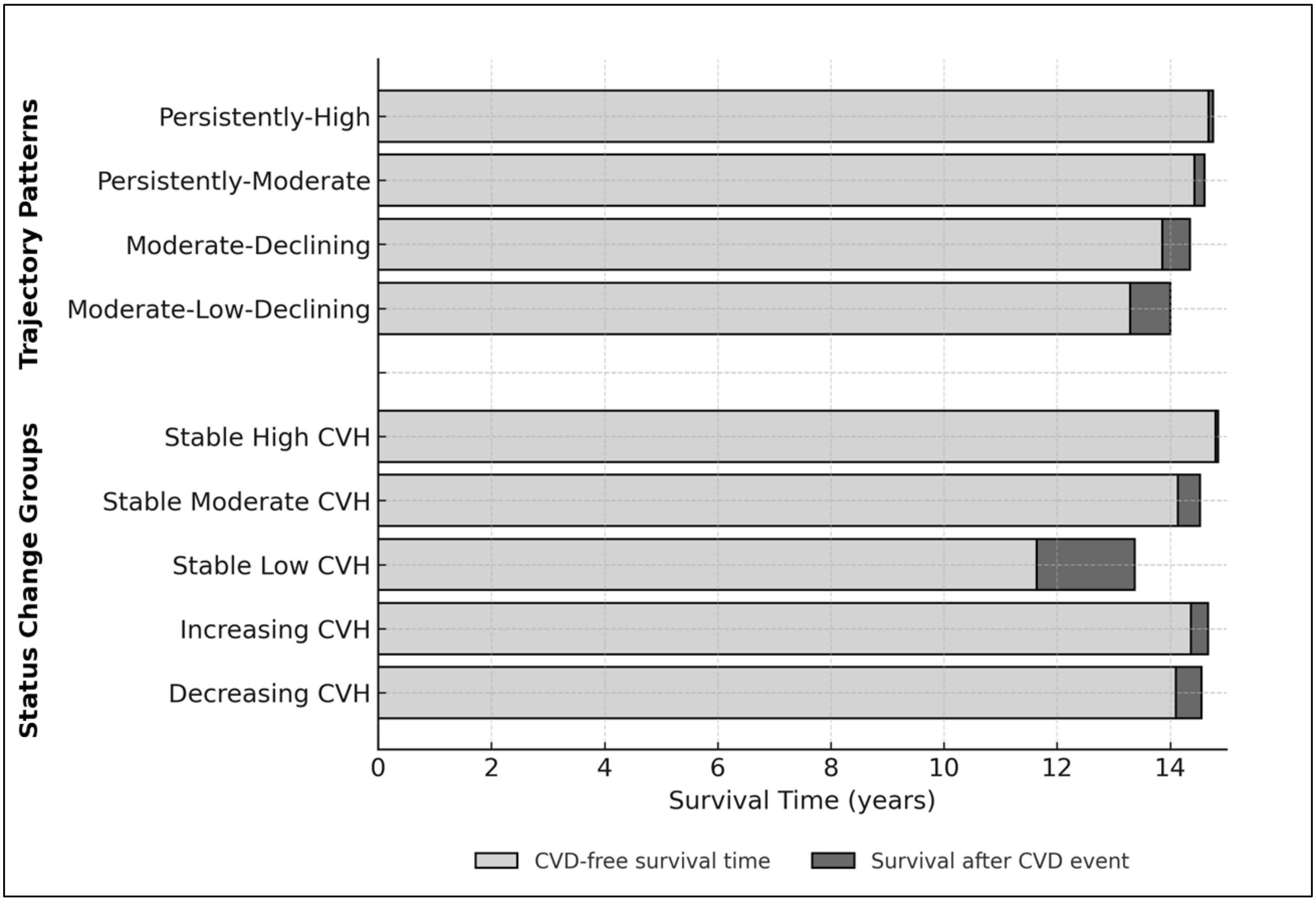

© 2025 Guo JW et al. *JAMA Network Open*.

eFigure 6. Distribution of Baseline LE8 Scores by CVH Status Change Groups

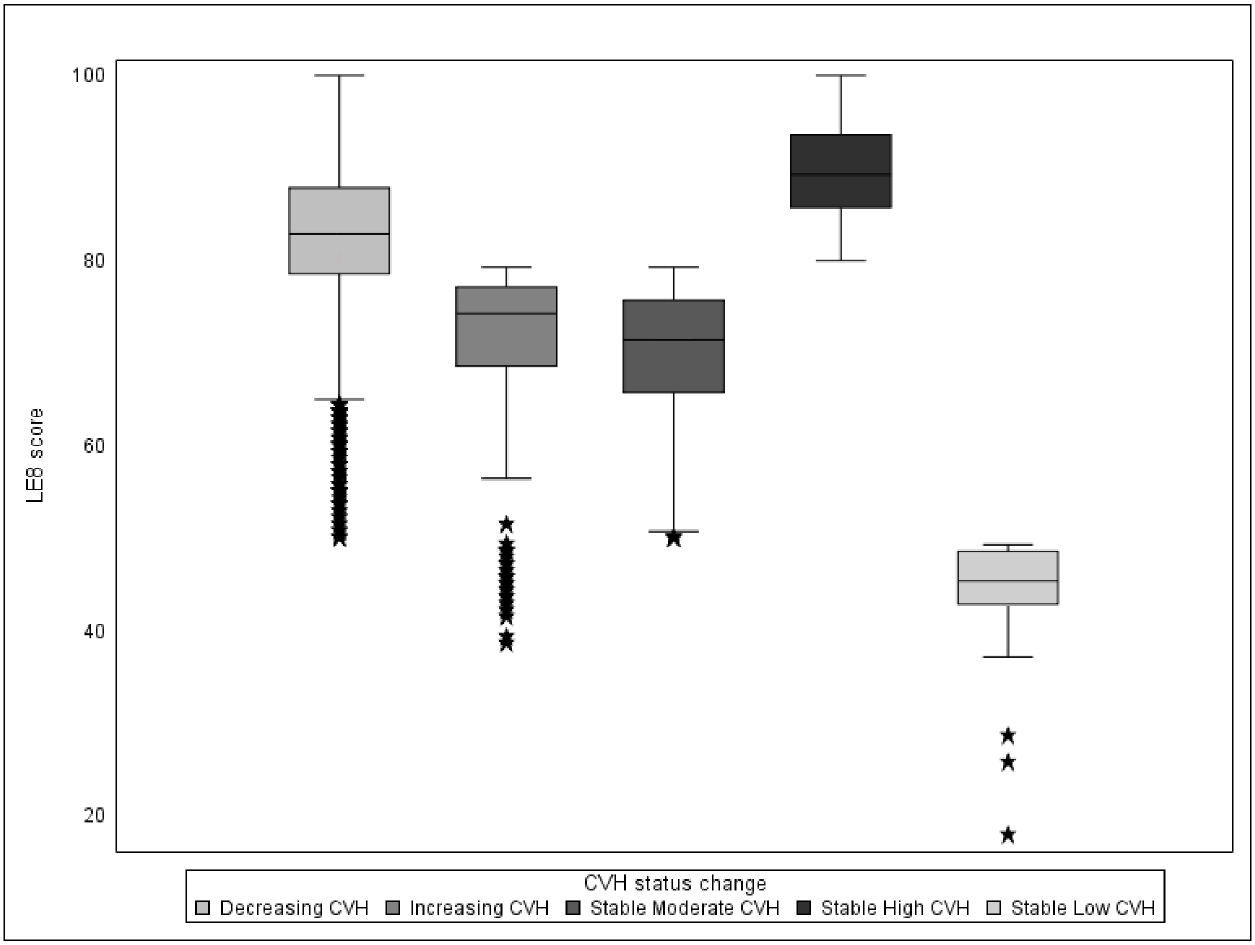

© 2025 Guo JW et al. *JAMA Network Open*.

eFigure 7. Event Incidence Rates by Status Change Groups

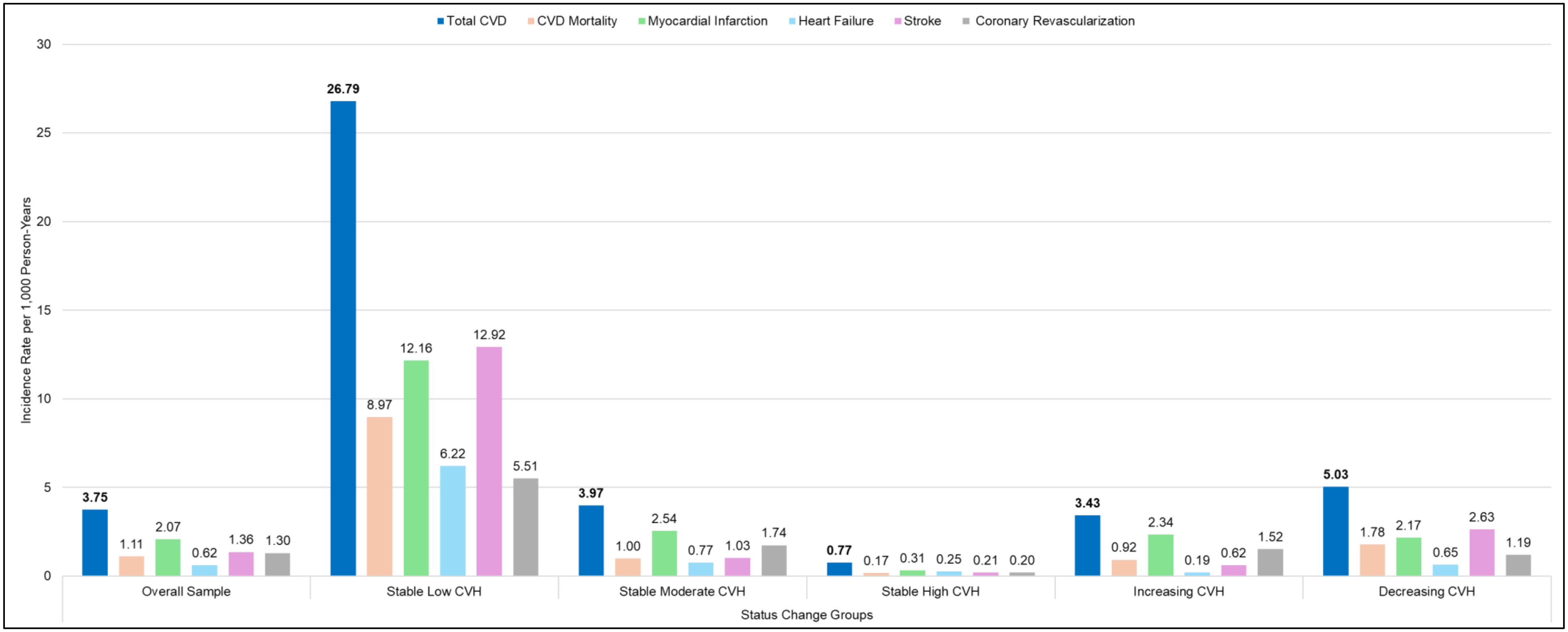

© 2025 Guo JW et al. *JAMA Network Open*.

eTable 1. Life's Essential 8 Score Components

CVH Metric	Method of Measurement	Quantification of CVH Metric	
Diet	Measurement: Self-reported daily intake on CARDIA Diet History questionnaire with quantiles of Healthy Eating Index 2015 scores	Points<br>100<br>80<br>50<br>25<br>0	Quantile<br>≥95th %ile (top/ideal diet)<br>75th – 94th %ile<br>50th – 74th %ile<br>25th – 49th %ile<br>1st – 24th %ile (bottom/least ideal quartile)
Physical Activity	Measurement: Self-reported exercise type, intensity, and duration on CARDIA Physical Activity Questionnaire	Points<br>100<br>90<br>80<br>60<br>40<br>20<br>0	Minutes<br>≥ 150<br>120 – 149<br>90 – 119<br>60 – 89<br>30 – 59<br>1 – 29<br>0
Nicotine Exposure	Measurement: Self-reported use of cigarettes	Points<br>100<br>75<br>50<br>25<br>0	Status<br>Never smoker<br>Former smoker, quit ≥5 yrs<br>Former smoker, quit 1 – <5 yrs<br>Former smoker, quit <1 yr<br>Current smoker
Sleep Health	Measurement: Self-reported average hours of sleep per night	Points<br>100<br>90<br>70<br>40<br>20<br>0	Level<br>7 – <9<br>9 – <10<br>6 – <7<br>5 – <6 or ≥10<br>4 – <5<br><4
BMI	Measurement: Body weight (kg) divided by height squared (m^2)	Points<br>100<br>70<br>30<br>15<br>0	Level<br><25<br>25.0 – 29.9<br>30.0 – 34.9<br>35.0 – 39.9<br>≥40.0
Blood Lipids	Measurement: Fasting blood sample total and HDL-cholesterol with calculation of non-HDL cholesterol	Points<br>100<br>60<br>40<br>20<br>0	Level<br><130<br>130 – 159<br>160 – 189<br>190 – 219<br>≥220
Blood Glucose	Measurement: Fasting blood glucose (FBG) or hemoglobin A1c % (HbA1c %)	Points<br>100<br><br>60<br><br>40<br>30<br>20<br>10<br>0	Level<br>No history of diabetes and FBG <100 (or HbA1c < 5.7)<br>No diabetes and FBG 100 – 125 (or HbA1c 5.7-6.4) (Pre-diabetes)<br>Diabetes with HbA1c <7.0<br>Diabetes with HbA1c 7.0 – 7.9<br>Diabetes with HbA1c 8.0 – 8.9<br>Diabetes with Hb A1c 9.0 – 9.9 0<br>Diabetes with HbA1c ≥10.0
Blood Pressure	Measurement: Systolic and diastolic BP (mmHg)	Points<br>100<br>75	Level<br><120/<80 (Optimal)<br>120-129/<80 (Elevated)

© 2025 Guo JW et al. *JAMA Network Open*.

CVH Metric	Method of Measurement	Quantification of CVH Metric	
		50<br>25<br>0	130-139 or 80-89 (Stage I HTN)<br>140-159 or 90-99<br>≥160 or ≥100<br><br>Subtract 20 points if treated

© 2025 Guo JW et al. *JAMA Network Open*.

eTable 2. Included and Excluded CARDIA Participant Characteristics at Baseline **(A)** Trajectory Analysis and **(B)** Status Change Analysis

(A) Trajectory Analysis

Descriptive Variable	Excluded (n=874)	Included (n=4,241)
Age, years	24.5 (3.8)	24.9 (3.6)
Male, %	440 (50.5%)	1887 (44.5%)
Black, %	595 (68.3%)	2042 (48.1%)
Education, years	13.8 (2.4)	15.6 (2.6)
HEI Diet score	59.9 (9.2)	61.9 (9.4)
Physical Activity, mins/wk	417.6 (381.5)	410.1 (362.1)
Current smoker, %	356 (41.4%)	1188 (28.2%)
Body Mass Index, kg/m2	24.8 (5.6)	24.4 (4.9)
Total Cholesterol, mg/dL	177.0 (36.3)	176.7 (32.9)
High-density lipoprotein, mg/dL	52.5 (14.0)	53.3 (13.1)
Serum Glucose, mg/dL	85.5 (30.6)	82.0 (11.5)
Diabetes Treatment, %	9 (1.0%)	5 (0.1%)
Systolic Blood Pressure, mmHg	111.5 (11.5)	110.2 (10.8)
Diastolic Blood Pressure, mmHg	69.3 (10.4)	68.4 (9.4)
Hypertension Treatment, %	32 (3.7%)	83 (2.0%)

(B) Status Change Analysis

Descriptive Variable	Excluded (n=2,255)	Included (n=2,857)
Age, years	24.6 (3.7)	25.0 (3.6)
Male, %	1083 (48.0%)	1244 (43.5%)
Black, %	1339 (59.4%)	1298 (45.4%)
Education, years	14.6 (2.6)	15.9 (2.6)
HEI Diet score	60.9 (9.4)	62.1 (9.4)
Physical Activity, mins/wk	418.8 (371.2)	405.5 (360.8)
Current smoker, %	821 (37.0%)	723 (25.3%)
Body Mass Index, kg/m2	24.9 (5.5)	24.2 (4.7)
Total Cholesterol, mg/dL	176.3 (34.7)	177.1 (32.5)
High-density lipoprotein, mg/dL	52.7 (13.7)	53.5 (12.9)
Serum Glucose, mg/dL	83.5 (21.1)	81.9 (11.4)
Diabetes Treatment, %	9 (0.4%)	5 (0.2%)

© 2025 Guo JW et al. *JAMA Network Open*.

Descriptive Variable	Excluded (n=2,255)	Included (n=2,857)
Systolic Blood Pressure, mmHg	111.2 (11.2)	109.8 (10.7)
Diastolic Blood Pressure, mmHg	68.9 (10.0)	68.4 (9.3)
Hypertension Treatment, %	62 (2.7%)	53 (1.9%)

© 2025 Guo JW et al. *JAMA Network Open*.

eTable 3. Adjusted Hazard Ratios of Incident CVD Events by CVH Trajectory Patterns Excluding Individuals With Mean PPP <0.7[a]

Analysis	Covariate	N	CVD Events (Fatal and Non-fatal)[b]	
			Hazard Ratio (95% CI)	P-value
Trajectory	Trajectory Pattern			
	Moderate-Low-Declining	347	10.07 (4.70-21.60)	<0.001
	Moderate-Declining	1,223	5.38 (2.69-10.75)	<0.001
	Persistently-Moderate	1,435	2.19 (1.05-4.58)	0.038
	Persistently-High	842	Ref	Ref

[a] PPP = Posterior prediction probability
[b] CVD events are defined as myocardial infarction, heart failure, stroke, coronary revascularization, and cardiovascular death. Models adjusted for age, sex, race, and maximal education

© 2025 Guo JW et al. *JAMA Network Open*.

eTable 4. Distribution of Status Change Groups (Increasing vs Decreasing)

**Increasing (N=286)**		**Y20 CVH Status, N (%)**		
		Low	Moderate	High
**Y0 CVH Status**	Low	–	27 (9.4)	0 (0)
	Moderate	–	–	259 (90.5)
	High	–	–	–
**Decreasing (N=780)**		**Y20 CVH Status, N (%)**		
		Low	Moderate	High
**Y0 CVH Status**	Low	–	–	–
	Moderate	208 (26.7)	–	–
	High	19 (2.4)	553 (71.0)	–

© 2025 Guo JW et al. *JAMA Network Open*.

eTable 5. Incidence Rates of CVD Events Occurring During Follow-Up, by Trajectory Pattern

**Event Type**	**Event Incidence Rates During Follow-Up (per 1,000 person-years)**				
	**Overall Sample (N=4,241)**	**Moderate-Low-Declining (N=382)**	**Moderate-Declining (N=1,357)**	**Persistently-Moderate (N=1,599)**	**Persistently-High (N=903)**
**Total CVD**[a]	3.716	10.33	5.70	2.30	0.97
**CVD Mortality**[b]	1.29	4.45	1.61	0.91	0.21
**Myocardial Infarction**	1.87	4.63	2.82	1.34	0.32
**Heart Failure**	0.79	2.26	1.20	0.42	0.25
**Stroke**	1.41	4.78	2.10	0.65	0.39
**Coronary Revasc.**	1.20	2.59	1.82	0.92	0.22

[a] Total CVD includes myocardial infarction, coronary revascularization (non-elective), heart failure, stroke, or other fatal heart or atherosclerotic disease.
[b] For CVD death, underlying causes of death included coronary or other heart disease, stroke, or other definite atherosclerotic disease.
CI, confidence interval; CVD, cardiovascular disease; CVH, cardiovascular health; Revasc., revascularization

© 2025 Guo JW et al. *JAMA Network Open*.

eTable 6. Sensitivity Analysis: CVD Incidence by CVH Trajectories and CVH Status Change in Young Adulthood, by Sex

			CVD Events (Fatal and Non-fatal)[a]	
**Analysis**	**Covariate**	**Participants (N)**	**Events (N)**	**Crude Incidence Rate per 1,000 Person-Years**
Trajectory (N=4,241)	Trajectory Pattern[b]			
	Male	1,887		
	Moderate-Low-Declining	157	24	13.68
	Moderate-Declining	667	63	7.44
	Persistently-Moderate	754	38	3.41
	Persistently-High	309	1	0.54
	Female	2,354		
	Moderate-Low-Declining	225	21	8.06
	Moderate-Declining	689	38	4.08
	Persistently-Moderate	845	15	1.33
	Persistently-High	595	11	1.19
CVH Status Change (N=2,857)	Status Change Group[b]			
	Male	1,244		
	Stable Low CVH	10	4	39.41
	Stable High CVH	183	1	0.40
	Increasing CVH[c]	98	8	5.94
	Decreasing CVH[c]	352	27	5.96
	Stable Moderate CVH	601	37	4.72
	Female	1,613		
	Stable Low CVH	16	3	19.48
	Stable High CVH	356	6	0.97
	Increasing CVH[c]	147	5	2.15
	Decreasing CVH[c]	526	25	4.28
	Stable Moderate CVH	560	26	3.26

[a] CVD events are defined as myocardial infarction, heart failure, stroke, coronary revascularization, and cardiovascular death.
[b] Adjusted for age, sex, race, maximal education
[c] Increasing and Decreasing CVH groups defined as participants whose CVH crossed over ≥1 categories. CVH categories defined as: High CVH = 80–100, Moderate CVH = 50-79, Low CVH = 0-49

© 2025 Guo JW et al. *JAMA Network Open*.

eTable 7. Sensitivity Analysis: CVD Incidence by CVH Trajectories and CVH Status Change in Young Adulthood, by Race

			CVD Events (Fatal and Non-fatal)[a]	
**Analysis**	**Covariate**	**Participants (N)**	**Events (N)**	**Crude Incidence Rate per 1,000 Person-Years**
Trajectory (N=4,241)	Trajectory Pattern[b]			
	Black	2,042		
	Moderate-Low-Declining	262	26	9.59
	Moderate-Declining	848	69	5.77
	Persistently-Moderate	748	25	2.49
	Persistently-High	184	4	1.65
	White	2,199		
	Moderate-Low-Declining	120	19	11.95
	Moderate-Declining	508	32	5.59
	Persistently-Moderate	850	28	2.14
	Persistently-High	721	8	0.80
CVH Status Change (N=2,857)	Status Change Group[b]			
	Black	1,298		
	Stable Low CVH	15	2	20.81
	Stable High CVH	106	1	0.49
	Increasing CVH[c]	105	7	4.88
	Decreasing CVH[c]	399	35	6.57
	Stable Moderate CVH	673	33	3.71
	White	1,559		
	Stable Low CVH	11	5	35.37
	Stable High CVH	433	6	0.84
	Increasing CVH[c]	181	6	2.59
	Decreasing CVH[c]	381	17	3.47
	Stable Moderate CVH	553	30	4.28

[a] CVD events are defined as myocardial infarction, heart failure, stroke, coronary revascularization, and cardiovascular death.
[b] Adjusted for age, sex, race, maximal education
[c] Increasing and Decreasing CVH groups defined as participants whose CVH crossed over ≥1 categories. CVH categories defined as: High CVH = 80–100, Moderate CVH = 50-79, Low CVH = 0-49

© 2025 Guo JW et al. *JAMA Network Open*.

eTable 8. CVD Hazard Ratios of Incident CVD by CVH Trajectories with Adjustment by Baseline LE8 Score

		**CVD Events (Fatal or Non-fatal)**	
**Trajectory Pattern Name**	**Participants (N=4,257)**	**Adjusted* HR (95% CI)**	**P-value**
Moderate-Low-Declining	437	7.40 (2.51–21.83)	<0.001
Moderate-Declining	1,405	4.48 (1.93–10.40)	<0.001
Persistently-Moderate	1,549	2.11 (0.94–4.72)	0.0706
Persistently-High	866	Ref	Ref

*Adjusted for age, sex, race, maximal education, and baseline (Y0) LE8 score

© 2025 Guo JW et al. *JAMA Network Open*.

eTable 9. Individual LE8 Metric Scores at Years 0 and 20, by Status Change Group

**LE8 Metric Score**[a] Mean (SD)	**CVH Status Change Groups**											
	**Overall (N=2,857)**		**Stable Low CVH (N=26)**		**Stable Moderate CVH (N=1,225)**		**Stable High CVH (N=540)**		**Increasing CVH (N=286)**		**Decreasing CVH (N=780)**	
	**Y0**	**Y20**	**Y0**	**Y20**	**Y0**	**Y20**	**Y0**	**Y20**	**Y0**	**Y20**	**Y0**	**Y20**
Diet	41.4 (31.4)	39.9 (31.3)	22.1 (23.0)	16.2 (23.1)	29.7 (27.4)	32.2 (28.6)	63.5 (27.1)	63.6 (26.9)	34.9 (28.1)	54.8 (30.1)	47.5 (31.6)	30.8 (28.1)
Physical Activity	83.4 (31.6)	73.4 (38.7)	34.9 (39.9)	38.1 (43.1)	78.5 (35.1)	72.3 (39.0)	95.4 (14.3)	91.1 (23.4)	69.8 (40.0)	90.5 (23.2)	89.6 (24.7)	58.0 (43.2)
Nicotine Exposure	83.4 (31.6)	73.4 (38.7)	34.9 (39.9)	38.1 (43.1)	78.5 (35.1)	72.3 (39.0)	95.4 (14.3)	91.1 (23.4)	69.8 (40.0)	90.5 (23.2)	89.6 (24.7)	58.0 (43.2)
Sleep Health	63.1 (31.8)	76.4 (26.3)	37.2 (28.0)	54.0 (28.5)	55.5 (32.1)	73.3 (27.3)	74.7 (27.3)	87.9 (17.4)	52.0 (31.4)	86.9 (19.3)	72.0 (29.4)	70.4 (28.2)
BMI	85.6 (24.5)	60.3 (33.9)	37.9 (31.6)	30.8 (33.1)	80.7 (26.6)	52.5 (33.2)	98.0 (8.3)	86.7 (18.8)	87.8 (24.5)	78.3 (27.5)	85.6 (23.7)	48.6 (32.6)
Blood Lipids	80.7 (25.7)	73.0 (28.5)	39.5 (24.5)	42.6 (29.2)	75.6 (26.5)	69.1 (28.6)	90.7 (19.1)	88.5 (20.1)	75.3 (27.8)	84.8 (23.1)	85.1 (23.7)	65.0 (29.2)
Blood Glucose	98.8 (7.1)	82.4 (24.0)	88.1 (22.0)	48.7 (34.2)	98.5 (8.0)	80.1 (24.1)	99.8 (2.9)	94.7 (13.8)	99.0 (6.7)	93.6 (16.4)	99.1 (6.1)	74.4 (25.7)
Blood Pressure	90.5 (19.2)	80.3 (28.5)	70.0 (31.6)	51.7 (38.0)	87.8 (21.0)	76.7 (28.2)	95.7 (12.9)	95.9 (12.6)	89.0 (20.7)	93.2 (17.4)	92.3 (17.6)	71.4 (33.1)

[a]LE8 metric scores range from 0-100 points according to the thresholds defined in **eTable 1**.

© 2025 Guo JW et al. *JAMA Network Open*.

eTable 10. Incidence Rates of CVD Events Occurring During Follow-Up, by Status Change Group

**Event Type**	**Event Incidence Rates During Follow-Up (per 1,000 person-years)**					
	**Overall Sample (N=2,857)**	**Stable Low (N=26)**	**Stable Moderate (N=1,225)**	**Stable High (N=540)**	**Increasing (N=286)**	**Decreasing (N=780)**
**Total CVD[a]**	3.75	26.79	3.97	0.77	3.43	5.03
**CVD Mortality[b]**	1.11	8.97	1.00	0.17	0.92	1.78
**Myocardial Infarction**	2.07	12.16	2.54	0.31	2.34	2.17
**Heart Failure**	0.62	6.22	0.77	0.25	0.19	0.65
**Stroke**	1.36	12.92	1.03	0.21	0.62	2.63
**Coronary Revasc.**	1.30	5.51	1.74	0.20	1.52	1.19

[a] Total CVD includes myocardial infarction, coronary revascularization (non-elective), heart failure, stroke, or other fatal heart or atherosclerotic disease.
[b] For CVD death, underlying causes of death included coronary or other heart disease, stroke, or other definite atherosclerotic disease.
CI, confidence interval; CVD, cardiovascular disease; CVH, cardiovascular health; Revasc., revascularization

© 2025 Guo JW et al. *JAMA Network Open*.

eReferences

1. Liu K, Slattery M, Jacobs D, Jr., et al. A study of the reliability and comparative validity of the cardia dietary history. *Ethn Dis*. Winter 1994;4(1):15-27.
2. McDonald A, Van Horn L, Slattery M, et al. The CARDIA dietary history: development, implementation, and evaluation. *J Am Diet Assoc*. Sep 1991;91(9):1104-12.
3. Krebs-Smith SM, Pannucci TE, Subar AF, et al. Update of the Healthy Eating Index: HEI-2015. *J Acad Nutr Diet*. Sep 2018;118(9):1591-1602. doi:10.1016/j.jand.2018.05.021
4. Jacobs DR, Jr., Hahn LP, Haskell WL, Pirie P, Sidney S. Validity and Reliability of Short Physical Activity History: Cardia and the Minnesota Heart Health Program. *J Cardiopulm Rehabil*. Nov 1989;9(11):448-459. doi:10.1097/00008483-198911000-00003
5. Guo JW, Ning H, Allen NB, Reges O, Gabriel KP, Lloyd-Jones DM. Association of Cardiovascular Health in Young Adulthood with Long-Term Blood Pressure Trajectories. *Am J Hypertens*. Apr 26 2024;doi:10.1093/ajh/hpae047
6. Cutter GR, Burke GL, Dyer AR, et al. Cardiovascular risk factors in young adults. The CARDIA baseline monograph. *Control Clin Trials*. Feb 1991;12(1 Suppl):1S-77S. doi:10.1016/0197-2456(91)90002-4
7. Rubin DB, Schenker N. Multiple imputation in health-are databases: An overview and some applications. *Statistics in Medicine*. 1991;10(4):585-598. doi:https://doi.org/10.1002/sim.4780100410

© 2025 Guo JW et al. *JAMA Network Open*.
