# Supplementary material for: Cardiovascular Health Changes in Young Adults and Risk of Later-Life Cardiovascular Disease
Source: JAMA Netw Open. 2025 Oct 6;8(10):e2535573. doi: 10.1001/jamanetworkopen.2025.35573 (PMC12501802; doi:10.1001/jamanetworkopen.2025.35573)
Supplement: Supplement 2. — Data Sharing Statement [file jamanetwopen-e2535573-s002.pdf]

Data Sharing Statement

Guo. Cardiovascular Health Changes in Young Adults and Risk of Later-Life Cardiovascular Disease. *JAMA Netw Open*. Published October 06, 2025.
doi:10.1001/jamanetworkopen.2025.35573

Data
Data available: Yes
Data types: Deidentified participant data
How to access data: Requests for data access can be made to the CARDIA Coordinating Center via the CARDIA website: https://www.cardia.dopm.uab.edu/.
When available: With publication

Supporting Documents
Document types: None

Additional Information
Who can access the data: Researchers whose proposed use of the data has been approved
Types of analyses: For any purpose
Mechanisms of data availability: With a signed data access agreement
